# Assessment of Level of Awareness, Current State of Knowledge, and Skills on Endocrown Technique Among Senior Saudi Dental Students and Interns

**DOI:** 10.7759/cureus.49897

**Published:** 2023-12-04

**Authors:** Manal R Alammari, Awwadh A Alzahrani, Abdullah Y Alaidarous, Mohammed M Alnasiri

**Affiliations:** 1 Oral and Maxillofacial Prosthodontics, King Abdulaziz University Faculty of Dentistry, Jeddah, SAU; 2 Dentistry, King Abdulaziz University Faculty of Dentistry, Jeddah, SAU

**Keywords:** skill, knowledge, awareness, endocrown, interns, dental students

## Abstract

Background

Awareness and knowledge about the endocrown technique contribute to the delivery of quality dental care and restorative long-term success. Dentists must be aware and knowledgeable about endocrowns as they offer a conservative approach, aid in maintaining tooth integrity, and have become a promising option in use recently.

Aim

This study evaluated the current state of knowledge, and the understanding of the endocrown technique as post-endodontic management among senior undergraduate dental students and interns who have studied the theoretical and laboratory procedures of endocrown as part of the curriculum.

Materials and methods

This cross-sectional investigation was conducted through an online questionnaire consisting of 21 questions in three parts. The first part consisted of demographic information; the second encompassed knowledge and attitude; and the third part evaluated skills regarding endocrown technique. A total of 164 voluntary dental students and dental intern questionnaires were evaluated. Factor analysis was used to identify the domains for knowledge and skill.

Results

In this study, the knowledge and skills of undergraduate dental students toward endocrowns were assessed. The reliability statistics showed favorable internal consistency (Cronbach’s alpha values: 0.513 for knowledge and 0.729 for skill component 1). Significant correlations were noticed between knowledge component 1 and skill component 1 (r = 0.647, p < 0.001), knowledge component 1 and skill component 2 (r = 0.333, p < 0.001), and knowledge component 1 and skill component 3 (r = 0.260, p = 0.001). These findings emphasize the importance of evaluating students’ understanding of endocrown. Significant differences were found among intern participants, fifth- and sixth-year dental students in component 1 of the knowledge (p-value<0.001), and all skills components.

Conclusion

Dental students have varying levels of knowledge and skills related to endocrown. Gaps in knowledge and skills have been detected, which dictates intervention actions, including curricular changes and training workshops.

## Introduction

Indirect approaches can be used in the treatment of teeth that are challenging to restore by direct approaches or have substance loss at a level that cannot be restored. While post and core crown treatments were the most commonly performed indirect method in the past, the advancements in adhesive dentistry and conservative approaches in restorative dentistry have permitted the development of treatment means for the restoration of teeth with excessive substance loss [1]. Post-endodontic treatment management comprises restoration that must protect and preserve the lasting tooth structure while giving a pleasing aesthetic look, form, and decent function. The topmost purpose of such post-endodontic management is to offer minimally aggressive teeth preparation with maximum conservation of tissues surrounding the restored teeth [2]. Such an aim is needed to achieve the tooth-restoration complex that is mechanically stabilized and has an adequate surface for adhesion [3]. There are several choices that the practitioner can make to manage the post-endodontic restoration, such as post and cores, crown, and other methods based on varied situations. All the available possibilities are based on tooth structural integrity, patients’ aesthetic demand, and the condition for the remaining tooth structure [4]. The endodontically treated teeth have a higher risk of breakage than the vital teeth structure due to its drop in biomechanical strength [5]. Many variations happen in endodontically treated teeth, such as stiffness reduction, and a decrease in fracture resistance due to its loss of structural integrity related to dental caries, trauma, or extensive cavity preparation through treatment. The option of post-endodontic treatment management is influenced by the different forms of teeth involved, whether the tooth is anterior or posterior, and the amount of tooth structure remaining [6].

The introduction of adhesives in conservative dentistry and the effective development of dentine adhesives altered the way dentists manage endodontically treated teeth, which makes the use of radicular posts a less chosen option as long as there is sufficient surface area in tooth structure for adhesion [7]. Pissis (1995) introduced a novel technique in which he suggested a single-unit porcelain core and crown for conventional metal post and core replacement [7]. By 1999, Bindle and Mörmann had introduced the endocrown technique, which was based on the Pissis concept. They described it as an adhesive, full porcelain crown that is fixed to the endodontically treated teeth for the posterior [7]. The endocrown consists of a single unit with a circular butt-joint finish line and a central retention cavity inside the pulp chamber. The goal of the endocrown procedure is to achieve minimally invasive preparations and to conserve the existing tooth structure. In addition, the ceramic fabrication technology evolution in dentistry, which includes dental computer-aided-design and computer-aided-manufacturing CAD/CAM and high-quality adhesive cementation production, has enhanced the manufacturing ideal of higher biocompatibility and optimal mechanical properties of the ceramic endocrown [8-11]. There are various advantages to using endocrown over posts and core and crown placement. The endocrown method requires less time in tooth preparation and application, as well as fewer treatment visits by patients [12]. The aesthetic appearance of endocrown placement also gives practitioners a reason to choose it as a restorative modality. The adhesive restorative used in the endocrown method may decrease the chance of microorganism infiltration from the coronal part to the apical portion of the root canal system, thus improving the success rate of root canal treatments [13]. Endocrown treatment is indicated in cases with suitable pulp chamber depth. In the cases where posts are contraindicated due to a narrow and short canal, the endocrown method can be used where adhesion is not certain and tooth structure remains [13].

This survey-based study aimed to evaluate the level of awareness and the current state of knowledge and understanding of the endocrown technique as post-endodontic management in practice among undergraduate dental students and interns who are introduced to the theoretical and laboratory parts of studying endocrown.

The null hypothesis stated that the knowledge and skills of senior dental students and dental interns of endocrown were comparable. However, the alternative hypothesis suggested that knowledge and skills varied among undergraduates and dental interns in Saudi Arabia.

## Materials and methods

After obtaining ethical approval and permission from King Abdulaziz University’s Research Committee (REC: 106-05-23), this cross-sectional survey was conducted between August 2023 and October 2023. A pilot study was conducted to check the readiness of the questionnaire. Following this, the final version was sent. The survey was conducted through an online standard questionnaire with multiple choice questions sent as a Google Form. The students’ responses were recorded, analyzed for flaws, checked for completeness, and taken up for assessment.

Participants and design 

The sample of this survey study consisted of 164 senior dental students and intern dentists, including 69 female and 95 male participants. Among the participants, 33 were in their fifth year in dentistry, 52 were in their sixth year, and 79 were intern dentists. The participants’ identifying information was kept private, and participation in the survey was entirely voluntary. The Google Forms platform was used to generate the survey, and participants were sent the form link over social media.

Survey design

The survey involved three parts and a total of 21 questions. The first part pertained to the consent to participate in the survey and questions about the demographic data information of the participants, such as gender, age, and year of study. The second part of the survey included nine questions that assessed the knowledge levels of the participants. The last part of the survey included nine questions about the participants’ skills regarding endocrowns. 

Statistical analysis

The data collected in this study was analyzed and visualized using IBM SPSS version 27 (IBM Corp., Armonk, NY, USA). Simple descriptive statistics analysis was used to define the characteristics of the study variables, in the form of counts and percentages for the categorical and nominal variables and in terms of mean and standard deviation for the continuous variables. Reliability analysis including a model of Cronbach’s alpha was used to study the properties of measurement scales, the items that compose the scales, and the average inter-item correlation. Factor analysis was used to identify the domains for “knowledge” and “skill,” producing four factors for knowledge and three for skill. The questions involved in each factor are shown in Table 1. For the comparison of the domains to demographics, which represent two group means and more than two groups, an independent t-test, and one-way Analysis of variance (ANOVA), with a least significant difference (LSD) test as a post hoc test, were used. These tests were performed with the assumption of normal distribution. Otherwise, Welch’s t-test for two-group means and the Games-Howell test for multiple groups were used as an alternative for the LSD test. Finally, a conventional p-value of <0.05 was the criterion used to discard the null hypothesis.

**Table 1:** The questions involved in each factor

Knowledge	
Component 1	Have you ever heard of endocrown treatment? What are the advantages of endocrown over traditional dental crowns? How does the endocrown method compare with other restorative options for teeth that have undergone root canal treatment, such as traditional dental crowns or composite fillings? Which of the following influences the selection of endocrowns as a treatment option?
Component 2	In your opinion, which is more costly? When do you think endocrown treatment should be performed?
Component 3	How would you define the endocrown method? What is the long-term prognosis of endocrown treatment compared with other restorative options for teeth that have undergone root canal treatment?
Component 4	What is the purpose of endocrown treatment?
Skills	
Component 1	Which of the following is more suitable for occlusal reduction? Which of the following burs is used to eliminate undercut caused during access cavity preparation? What is the form of retention in endocrowns? Which of the following challenges could you encounter throughout the endocrown process?
Component 2	Can you name the following preparation technique in picture A? Can you name the following preparation technique in picture B?
Component 3	What type of radiograph should be taken to evaluate the adaptation of endocrowns? Have you had any hands-on experience with placing endocrown during your clinical training?

## Results

This study evaluated the level of awareness and the current state of knowledge and understanding of endocrown treatment as one post-endodontic management option in practice among undergraduate dental students who are introduced to the theoretical and procedural parts of studying endocrowns.

**Figure 1:**
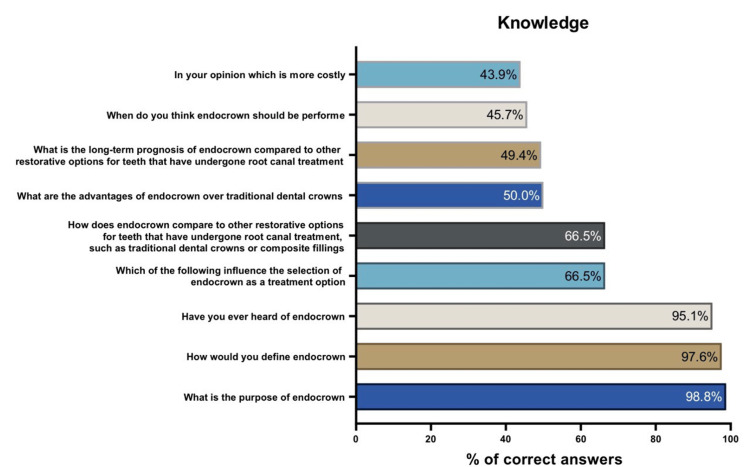
Percentage of correct answers to knowledge questions

**Figure 2:**
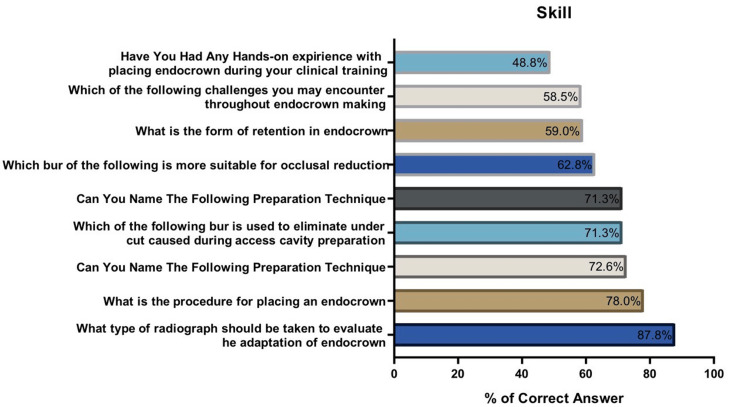
Percentage of correct answers to skill questions

Figures 1, 2 show the percentage of correct answers to the knowledge and skills questions regarding endocrown treatment. As shown in Figure 1, the weakest knowledge was on which option is more costly, while the dental student’s strongest knowledge was their knowledge on the purpose of endocrown treatment. On the other hand, Figure 2 illustrates that the weakest skill of dental students regarding endocrown treatment was related to their hands-on experience with placing an endocrown during their clinical training, while the strongest skill was their capacity to identify what type of radiograph should be taken to evaluate the adaptation of an endocrown.

Table 2 shows the factor analysis results for the knowledge of commonalities among senior dental students and interns. The results showed similar initial values of 1.000 for all commonalities. Consequently, they revealed the highest extraction value of 0.777 for the “purpose of endocrown” and the lowest extraction value of 0.421 for the “preferred time for endocrown performance” commonalities. The total variance explained based on eigenvalues was found to be half above the average value of 58.93. For the factor analysis toward the pattern matrix, the highest value of 0.861 was observed for the “purpose of endocrown” matrix under component 4, while the lowest value of −0.676 for the “hearing of or being familiar with endocrowns” matrix under component 2 (Table 3).

**Table 2:** Factor analysis of knowledge among participants

Communalities	Initial	Extraction
What are the advantages of endocrowns over traditional dental crowns?	1.000	0.564
How does endocrown treatment compare with other restorative options for teeth that have undergone root canal treatment, such as traditional dental crowns or composite fillings?	1.000	0.557
Which of the following influences the selection of endocrown as a treatment option?	1.000	0.434
When do you think endocrown should be performed?	1.000	0.421
In your opinion, which is more costly?	1.000	0.670
Have you ever heard of endocrown?	1.000	0.575
How would you define endocrown?	1.000	0.672
What is the purpose of endocrown?	1.000	0.777
What is the long-term prognosis of endocrown treatment compared with other restorative options for teeth that have undergone root canal treatment?	1.000	0.633
Total Variance Explained based on Eigenvalues 58.93		

**Table 3:** Factors analysis knowledge towards its commonalities of the participants Extraction method: principal component analysis, Rotation method: oblimin with kaiser normalization.

Pattern Matrix^a^	Component			
	1	2	3	4
What are the advantages of endocrowns over traditional dental crowns?	0.735			
How does endocrown treatment compare with other restorative options for teeth that have undergone root canal treatment, such as traditional dental crowns or composite fillings?	0.655			
Which of the following influences the selection of endocrown as a treatment option?	0.571			
When do you think endocrown treatment should be performed?	0.428	-0.419		
In your opinion, which is more costly?		0.806		
Have you ever heard of endocrown?		-0.676		
How would you define endocrown?			0.807	
What is the purpose of endocrown?				0.861
What is the long-term prognosis of endocrown treatment compared with other restorative options for teeth that have undergone root canal treatment?			0.447	-0.544

As Table 4 shows, the factor analysis results for skills commonalities among the dental students showed similar initial values of 1.000 for all commonalities, and revealed the highest extraction value of 0.745 for the “hands-on experience with placing endocrowns during clinical training” and the lowest extraction value of 0.305 for the “challenges that may be encountered throughout endocrown making” component. The total variance explained based on eigenvalues was found to be half above the average value of 58.44. For the factor analysis toward the pattern matrix, the highest value of 0.876 was observed for the “hands-on experience with placing endocrown during the clinical training” matrix under component 3, while the lowest value of −0.508 was found with the “procedure for placing an endocrown” matrix, also under component 3 (Table 5).

**Table 4:** Factor analysis of skills among participants

Communalities	Initial	Extraction
Which bur of the following is more suitable for occlusal reduction?	1.000	0.634
Which of the following burs is used to eliminate undercut caused during access cavity preparation?	1.000	0.545
What is the form of retention in endocrown treatment?	1.000	0.592
Which of the following challenges might you encounter throughout the endocrown process?	1.000	0.305
What is the procedure for placing an endocrown?	1.000	0.599
Can you name the following preparation technique A?	1.000	0.526
Can you name the following preparation technique B?	1.000	0.724
What type of radiograph should be taken to evaluate the adaptation of endocrown?	1.000	0.589
Have you had any hands-on experience with placing endocrowns during your clinical training?	1.000	0.745
Total Variance Explained based on Eigenvalues 58.44		

**Table 5:** Factor analysis of skills on pattern matrix among participants Extraction method: principal component analysis, Rotation method: oblimin with Kaiser normalization.

Pattern Matrix	Component		
	1	2	3
Which of the following bur is more suitable for occlusal reduction?	0.819		
Which of the following burs is used to eliminate undercut caused during access cavity preparation?	0.758		
What is the form of retention in endocrowns?	0.747		
Which of the following challenges might you encounter throughout the endocrown process?	0.528		
Can you name the following preparation technique?		0.848	
Can you name the following preparation technique?		0.671	
What type of radiograph should be taken to evaluate the adaptation of endocrowns?		0.633	0.444
Have you had any hands-on experience with placing endocrowns during your clinical training?			0.876
What is the procedure for placing an endocrown?	0.430		−0.508

The reliability statistics analysis on the knowledge and skills of the dental students regarding endocrown treatment showed favorable Cronbach alpha values of 0.513 (N = 4) for component 1 under knowledge, 0.729 (N = 5) for component 1, and 0.559 (N = 3) for component 2 under the skill domain (Table 6). This indicates the validity, internal consistency, and reliability of the skill and knowledge measurements among the students.

**Table 6:** Reliability statistics on the knowledge and skills towards endocrown of the participants

Reliability Statistics	Cronbach’s Alpha	N of Items
Knowledge		
Component 1	0.513	4
Component 2	−0.480	3
Component 3	0.083	2
Component 4	−0.099	2
Skills		
Component 1	0.729	5
Component 2	0.559	3
Component 3	−0.397	3

Table 7 shows the correlation among the components of skill and knowledge on endocrown treatment of the dental students using a two-tail test at a level of 0.01 (N = 164). The results showed a significant correlation between knowledge component 1 and knowledge component 2 (r = 0.401, p < 0.001), skill component 1 (r = 0.647, p < 0.001), skill component 2 (r = 0.333, p < 0.001), and skill component 3 (r = 0.260, p = 0.001). A significant correlation was also observed between knowledge component 2 and skill component 1 (r = 0.297, p < 0.001), as well as between knowledge component 3 and knowledge component 4 (r = 0.934, p < 0.001), and between skill component 2 and skill component 3 (r = 0.433, p < 0.001). In addition, skill component 1 was found to have a significant correlation with skill component 2 (r = 0.332, p < 0.001) and skill component 3 (r = 0.288, p < 0.001).

**Table 7:** Correlation among the components of skill and knowledge on endocrown treatment of the participants (N = 164) **. Correlation is significant at the 0.01 level (2-tailed).

Correlations		Knowledge Component 2	Knowledge Component 3	Knowledge Component 4	Skill Component 1	Skill Component 2	Skill Component 3
Knowledge Component 1	r	0.401**	-0.065	-0.134	0.647**	0.333**	0.260**
	p-value	<0.001	0.408	0.086	<0.001	<0.001	0.001
	N	164	164	164	164	164	164
Knowledge Component 2	r		0.013	-0.037	0.297**	0.084	0.010
	p-value		0.867	0.635	<0.001	0.287	0.902
	N		164	164	164	164	164
Knowledge Component 3	r			0.934**	-0.071	0.007	0.066
	p-value			<0.001	0.364	0.925	0.402
	N			164	164	164	164
Knowledge Component 4	r				-0.118	-0.055	0.008
	p-value				0.131	0.486	0.916
	N				164	164	164
Skill Component 1	r					0.332**	0.288**
	p-value					<0.001	<0.001
	N					164	164
Skill Component 2	r						0.433**
	p-value						<0.001
	N						164

Finally, as shown in Table 8, the levels of knowledge and skills on endocrown treatment were higher among the intern participants than in the fifth- and sixth-year dental students. Significant differences were found between them in component 1 of the knowledge and skills matrix.

**Table 8:** Skills and knowledge in relation to the respective years of dentistry A-significant using one-way analysis of variance (ANOVA) test at <0.05 level, B-post-hoc test (LSD test), C-post-hoc test (Games–Howell test)

Year	Total	5th year	6th year	Intern	p-value
Knowledge					
Component 1	164	1.55 ± 1.1^A^	2.12 ± 1.1^B^	2.71 ± 1.2^C^	<0.001^a,b^
Component 2	164	1.76 ± 0.7	1.98 ± 0.6	1.80 ± 0.6	0.186
Component 3	164	1.42 ± 0.6	1.50 ± 0.5	1.47 ± 0.6	0.819
Component 4	164	1.45 ± 0.5	1.50 ± 0.5	1.48 ± 0.5	0.921
Skills					
Component 1	164	2.67 ± 1.6^A^	3.29 ± 1.7^AB^	3.54 ± 1.6^B^	0.031^a,b^
Component 2	164	1.79 ± 1.0^A^	2.17 ± 0.9^A^	2.63 ± 0.7^B^	<0.001^a,c^
Component 3	164	1.73 ± 0.6^A^	2.04 ± 0.7^B^	2.39 ± 0.5^C^	<0.001^a,b^

## Discussion

There is a great deal of discussion in the dentistry literature about the restoration of teeth that have undergone endodontic treatment. Endodontically treated teeth carry a higher risk of biomechanical failure than vital teeth and are a common problem in restorative dentistry due to the fractures occurring in such teeth [2,14]. Rather than dehydration or physical changes in dentin, the loss of structural integrity associated with stress, trauma, and excessive cavity preparation is the main cause of a reduction in stiffness and the fracture resistance of teeth that have undergone endodontic treatment. To provide the solid basis needed for restoration and to increase the structural strength of the restored tooth, the quality and integrity of the remaining tooth structure should be carefully preserved [14,15]. According to biomechanical principles, a tooth’s structural strength is determined by the amount and intrinsic strength of its hard tissues, as well as the integrity of the anatomic form [16]. The present study aimed to assess the level of knowledge, skills, and understanding among senior undergraduate dental students and interns regarding endocrown treatment as a post-endodontic management technique.

As a result of developments in cementation methods, adhesive techniques, and dental materials utilized for tooth restoration, the outlook for inlay, onlay, and endocrown restorations has improved significantly [17]. Because they preserve dental tissue and, consequently, their long-term prognosis, these restorations, with their monoblock construction-are appropriate for conservative therapy [18]. The restoration process for teeth in the posterior area also varies based on the tooth that is to be treated [19]. It was stated that the size of the pulp chamber directly correlates with the performance of endocrown restorations in posterior teeth under axial and lateral stresses during functioning [20]. Other factors that affect the planning of restorations are the remaining tooth structure and functional requirements [21].

The pulpal walls offer macromechanical retention for the endocrown, which is attached to the pulp chamber’s interior and the cavity borders. Adhesive cementation is used to achieve micromechanical retention. This approach is especially useful when there is significant crown tissue loss and little interproximal space, and when typical post-and-crown rehabilitation is not achievable due to insufficient ceramic thickness [22]. Compared with traditional crowns, endocrowns are easier to place and take less clinical time. In addition, endocrowns have several benefits, including low cost, quick preparation time, simplicity of application, little chair time, and good aesthetic properties [23]. The findings in this study revealed interesting insights into students’ knowledge and skills related to the endocrown technique. Figure 1 depicts the distribution of correct answers for the knowledge-related questions. The weakest aspect of knowledge was identified as the perception of cost, whereas the students demonstrated a strong understanding of the purpose of endocrown placement.

Endocrown preparation involves creating the crown and core as a single-unit monoblock construction without using the root canal for support. It comprises a central retention cavity inside the pulp chamber and a circumferential 1.0-1.2 mm depth butt margin utilizing a green diamond wheel bur to reduce the occlusal surface. The shape of the diamond wheel bur enables precise control over the orientation of the reduction and facilitates the creation of a flat surface [7,10]. According to Mörmann et al., endocrowns with an occlusal thickness of 5.5 mm have two times the fracture resistance of ceramic crowns with a conventional preparation and an occlusal thickness of 1.5 mm [24]. According to Bindl and Mörmann’s evaluation of the performance of 208 endocrowns cemented to premolars and molars, the premolars exhibited more failures than the molars due to adhesion loss [25]. The lower adhesive bonding surface of premolars compared with that of molars is thought to be the reason for the loss of endocrown adhesion. In addition, premolars may have more leverage than molars because of their larger prepared tooth structure to crown ratio [26].

Endocrowns have exhibited impressive survival rates, ranging from approximately 86.9% to 90.5% to 99% relative to traditional crowns [27-29]. These favorable outcomes align with the findings of previous studies, which reported survival rates of 82.3% [30], 88% [8], and 87.1% [PR1] [25]. Regarding the skills, attitude, and confidence toward endocrowns, Figure 2 illustrates the distribution of skill-related answers, with the weakest skill observed in the students’ practical experience of placing endocrowns during clinical training. On the other hand, their ability to identify the appropriate radiograph type for evaluating endocrown adaptation was identified as their strongest skill.

Factor analysis was conducted to explore the commonalities among the dental students’ knowledge (Table 2) and skills (Table 4). The results indicated that the purpose of endocrown treatment had the highest factor extraction value (0.777) for knowledge, while the preferred time for endocrown performance showed the lowest extraction value (0.421). In terms of skills, hands-on experience with placing endocrowns during clinical training demonstrated the highest extraction value (0.745), whereas challenges encountered throughout endocrown fabrication exhibited the lowest extraction value (0.305). Reliability statistics, as indicated by Cronbach’s alpha values, were favorable for both the knowledge and the skills domains, confirming the validity, internal consistency, and reliability of the measured skills and knowledge among the students.

Table 8 shows the difference in skill and knowledge in relation to years of dentistry. The p-values associated with each component and skill indicate the level of statistical significance, further supporting the notion that more experience leads to notable variations in performance. The statistical tests conducted in Table 8, such as the post hoc tests and the one-way ANOVA Test, provide further evidence of the significance of these differences. Overall, the findings demonstrate how experience positively affects the knowledge and skills being evaluated. Over time, students tend to gain more information, skills, and competencies, which enhances the dentist’s ability to make a judgment and the correct clinical decision [31,32]. These results highlight the value of obtaining practical expertise and how it may improve performance and proficiency in various areas.

The current study has certain limitations. First, our results might not reflect the overall knowledge and attitudes of dental students in the country as the study was conducted in only one university in Saudi Arabia, albeit one of the largest governmental universities in the country. Second, a limited sample size was evaluated. To deliver an even better outcome in the future, a bigger sample size should be obtained and more of the main universities included. Finally, the higher response rate among interns might be explained by the situation of the three authors, who are at the same educational level, which may have encouraged any unenthusiastic respondents to participate.

## Conclusions

The study findings suggest that senior dental students and dental interns possess varying levels of knowledge and skills related to endocrown placement. Understanding the students’ grasp of endocrown concepts collectively is vital for evaluating their overall ability in this post-endodontic management procedure. In assessing the level of awareness, current knowledge, and understanding of future dentists, it is crucial to focus on the limitations of the present curriculum, which dictates intervention actions, including curricular modification and training workshops.
